# Mono-Causal and Multi-Causal Theories of Disease: How to Think Virally and Socially about the Aetiology of AIDS

**DOI:** 10.1007/s10912-017-9441-9

**Published:** 2017-04-04

**Authors:** Katherine Furman

**Affiliations:** 1 Department of Philosophy, University of Durham, Durham, UK; 2 London School of Economics and Political Science, London, UK

**Keywords:** Disease aetiology, Ontology, Mono-causal/multi-causal theories, South Africa, AIDS denialism, Thabo Mbeki

## Abstract

In this paper, I utilise the tools of analytic philosophy to amalgamate mono-causal and multi-causal theories of disease. My aim is to better integrate viral and socio-economic explanations of AIDS in particular, and to consider how the perceived divide between mono-causal and multi-causal theories played a role in the tragedy of AIDS denialism in South Africa in the early 2000s. Currently, there is conceptual ambiguity surrounding the relationship between mono-causal and multi-causal theories in biomedicine and epidemiology. Mono-causal theories focus on single, typically microbial, sources of illness and are most concerned with infectious diseases. By contrast, multi-causal theories allow for multiple factors to underpin a disease’s aetiology, including socio-economic and behavioural factors, and they usually focus on chronic non-communicable diseases. However, if these theories are taken to be strictly distinct, this prevents the inclusion of both microbial and socio-economic factors in a single explanation of any particular disease. This strict distinction became a problem when trying to explain the disproportionate prevalence of AIDS in southern Africa and ultimately contributed to the tragedy of AIDS denialism in South Africa. In tandem with viewing how the perceived divide between multi-causal and mono-causal theories underpinned AIDS denialism, I examine Thabo Mbeki’s specific role, while acknowledging that AIDS is being deprioritised on a broader international level. Overall, I will demonstrate that any long-term plan to eliminate AIDS will require viral and socio-economic factors to be considered simultaneously and that such a theoretical approach requires a clearer understanding of the underlying concepts of disease aetiology.

## Introduction

AIDS is taking on diminished importance in the international political agenda as we enter the ‘post-AIDS’ era. In particular, this can be seen in how AIDS is addressed in the new United Nations Sustainable Development Goals (SDGs), launched as benchmark goals across the UN system in 2015 with a fifteen-year roadmap set for 2030. These SDGs replace the previous Millennium Development Goals (MDGs) which were launched in 2000 and expired in 2015. Within the MDGs, AIDS was explicitly foregrounded in goal 6 which aimed ‘to combat HIV/AIDS, Malaria and Other Diseases.’ However, AIDS is now listed as a subsidiary target of a much broader goal, aimed at promoting ‘Good Health and Well-Being’ (United Nations General Assembly 2010; United Nations General Assembly 2015). Part of what motivates the deprioritising of AIDS in particular is the understanding, as Alan Whiteside notes, that we now know what causes the disease, how it is transmitted, and how to treat it. More specifically, we now have excellent technical knowledge of viral aspects of AIDS, and there is a common sense conception that treatment availability corresponds to the end of the epidemic (Whiteside 2015, 460). However, this improvement in viral understanding has not brought an end to the disease, and it remains disproportionately prevalent in eastern and southern Africa (Whiteside 2015, 458). Any long-term solution requires that socio-economic drivers and viral aspects of the disease be considered simultaneously, and it is important that the underlying conceptual framework can support this programme.

In 1986, the scientific community moved from multiple theories of the possible aetiology of AIDS to accepting HIV as the definitive cause. While this was aetiologically rational, it resulted in a loss of some explanatory power. There were aspects of the disease that could not easily be explained in strictly viral terms but which seemed to require reference to elements of the now discarded ‘immune overload’ theory: the theory that AIDS is due to diminished immune function resulting from various ‘lifestyle related’ factors such as multiple STD infections, drug use, and malnutrition. For instance, at least part of the disproportionate prevalence of AIDS in southern Africa could be explained by drawing on the idea that malnutrition, and already having been exposed to multiple other infections, places strain on the immune system and, thus, some individuals become more susceptible to opportunistic infections. Mosley makes the point as follows:

It has been estimated that in America, the odds of contracting AIDS from an infected heterosexual partner is 1 in 500. But in many parts of Africa, the odds are 1 in 10. This difference in susceptibility to infection is explained, not by reference to HIV, nor to commensurably higher rates of unprotected sex that can be corrected by a focus on sex education. People suffering from malnutrition, parasites, and other forms of illness have compromised immune systems that make them more susceptible to infection by HIV than comparable healthy, well-nourished individuals in industrialised countries (2004, 409).^1^

While Mosley’s point is controversial (more will be said about this later), it is clear that one can be committed to a disease being viral and still draw on various socio-economic factors to explain aspects of that disease. However, being committed to a disease as both microbially-caused and subject to socio-economic factors becomes difficult because of an ambiguity in the relationship between mono- and multi-causal theoretical accounts of disease in biomedicine and epidemiology. These two approaches are often taken to explain different types of disease, where mono-causal accounts are used to explain infectious diseases and multi-causal accounts are used to explain chronic non-communicable disease (diabetes, hypertension, heart disease, certain forms of cancer, etc.). This is a problem in the case of AIDS because the available conceptual framework for understanding disease requires that one focus on either viral or socio-economic factors but not both.

Turning to the South African context, the strict division between mono-causal and multi-causal theoretical accounts of disease provides a plausible (if charitable) account of former South African President, Thabo Mbeki’s, AIDS denialism in the early 2000s.^2^ On this description, Mbeki wanted an explanation for why social issues, such as poverty, seemed to be so intertwined with AIDS. The mainstream viral research programme at the time (a strictly mono-causal account) failed to provide an adequate explanation, which led Mbeki to consult non-mainstream AIDS scientists (who were very multi-causal in their approach). The non-mainstream scientists were able to provide a plausible-sounding account of the link between poverty and AIDS but also incorrectly rejected the causal role of the virus in the process.

Given the scale of the tragedy of AIDS denialism in South Africa—best estimates indicate that it resulted in 171,000 new HIV infections and 343,000 deaths between 1999 and 2002 (Nattrass 2008, 157)—this should put pressure on us to resolve the mono-causal/multi-causal divide, in order to find a way to incorporate multiple causal factors while still maintaining the causal salience of the virus. Susser’s (1973) layered multi-causal model offers a solution to this problem but raises a number of philosophical issues in the process. In particular, it introduces a new ambiguity related to the ontological status of the levels in his account and the relationship between those levels (Russo 2011, 77). I argue that this is not a serious problem for Susser’s account because he should not be understood as providing a metaphysical account of the status of levels. Rather, he is making a methodological point about how epidemiologists should think about disease in order to make progress in their day-to-day research programme.

The conceptual problems created by too strict a division between mono-causal and multi-causal accounts of disease are not restricted to the case of AIDS but extend to all microbially-based diseases that also have a social component. Stillwaggon’s comments on the outbreak of plague in Europe are particularly instructive in this regard:

Throughout history it has been clear that the epidemic spread of disease requires favorable conditions. Rats (or soldiers) aboard a ship from an eastern port carried plague-infected fleas into Italy in 1348 and sparked the epidemic spread of plague in Europe, wiping out one-third of the population in most of the continent. This introduction was a random event, but it was certainly not Western Europe’s only exposure to rats or plague. In 1348, plague entered a continent weakened by 30 years of falling per capita food consumption and increasing immiseration of the peasantry due to increased feudal demands. The population of Europe had already been falling in the decades leading up to 1348, and a series of disastrous harvests exacerbated the effects of war… Even though many nobles and townspeople perished in the Black Death, the ecologic context for the epidemic was the worsening economic situation of the peasantry. (2006, 8)

As Stillwaggon’s plague example makes clear, there are some ways in which this is old news. Practitioners of epidemiology and the biomedical sciences have been aware for many years that both infectious agents and socio-economic factors must be considered simultaneously to make sense of disease. However, the AIDS case makes it clear that this historical lesson has not been taken to heart, sometimes with tragic consequences, and attention needs to be paid to this theoretical concern.

## Mono-causal and multi-causal theoretical approaches to disease

The mono-causal account of disease came into existence in the nineteenth century. Prior to that, the miasma theory (the theory that diseases, such as cholera, arose due to poor air quality) and the humour theory (that disease came about when the four fluids of the body—black bile, yellow bile, blood and phlegm—became unbalanced) dominated (Lee 2012, 117; Codell Carter 2003, 10).

In the 1860s the germ theory of disease came into being when Louis Pasteur identified that microbes were involved in fermentation. Realising that something microbial could cause fermentation and the ability to identify microbial agents for the first time opened up the conceptual space in which it became conceivable that microbes could be involved in all kinds of biological processes (this is often referred to as the “age of bacterial discovery”) (Evans 1993, 8–9). This heralded in the mono-causal account of disease (Thagard 1999, 24–25).

In its broadest construal, the mono-causal account is just the insistence “that every disease has one cause that is necessary and, in limited circumstances, sufficient for disease” (Broadbent 2009, 302).^3^ Nobody, of course, thought that any disease was literally mono-causal, given that no event is ever actually the product of only a single cause (Lee 2012, 134). Broadbent goes on to clarify that what is really meant is that there is a single cause that meets the criteria of Koch’s Postulates—the guidelines that scientists use to establish causation between an identified microbe and the disease it is suspected to produce (Broadbent 2013, 149).

Adopting the mono-causal approach proved to be a very fruitful research programme. On discovering a new disease, the practical task became one of identifying a plausible microbial causal agent and then using Koch’s Postulates to establish causation. This approach allowed for the identification of the microbes responsible for a wide range of diseases (Koch himself, in the nineteenth century, identified the microbial agents responsible for cholera, tuberculosis and anthrax) and the discovery of pasteurisation and immunisation (Lee 2012, 117; Evans 1993, 10).

Despite the early successes of the mono-causal approach, the multi-causal account was developed as a competitor to it in the mid-twentieth century. Increased incidence of chronic non-communicable diseases (CNCDs) (diabetes, heart disease, certain forms of cancer, hypertension—diseases typically associated with lifestyle) could not be explained by reference to a single salient causal factor (Broadbent 2009, 305). The multi-causal model came to be identified with the central metaphor of the ‘web of causation,’ which Krieger describes as follows:

Conceptually, the metaphor evoked the powerful image of a spider’s web, an elegantly linked network of delicate strands, the multiple intersections representing specific risk factors or outcomes, and the strands symbolizing diverse causal pathways. It encouraged epidemiologists to look for multiple causes and multiple effects, and to identify the many—as opposed to singular—routes by which disease could be prevented. (1994, 891)

A more recent development in multi-causalism has been the introduction of syndemics in the early 2000s—initially the idea that diseases interact with each other to exacerbate the effects of the individual diseases, but later also that diseases interact with social and environmental factors to similar effect (Singer and Clair 2003). This is a straightforwardly multi-causal account of disease and is subject to the same benefits and criticisms as the multi-causal approach more generally.

Two plausible theories of disease are now available to us: the mono-causal account, which was developed to explain infectious diseases, and the multi-causal ‘web’ which came about in the middle of the twentieth century to explain CNCDs. It is not immediately clear what the relationship between these accounts of disease is, or should be.

In the case of AIDS, practising scientists working on the disease in the 1990s were, in their methodologies, strongly committed to mono-causalism. Robert Gallo, one of the scientists credited with the discovery of HIV, explicitly states that he is committed to the position that “… multifactorial is multi-ignorance. Most of the factors go away when we learn the real cause of a disease” (1991, 148). This quote is taken from his autobiography in a chapter entitled “A Single Disease with a Single Cause” —a title that further emphasises Gallo’s commitment to mono-causalism. Oppenheimer (1992) argues that the clinical isolation of HIV resulted in a substantial decrease in the number of epidemiological AIDS studies that were undertaken in the 1990s (49–50). Given that epidemiology is the multi-causal health science *par excellence*, this is another indicator that the mono-causal account had taken centre-stage in AIDS research as the viral research programme grew in the late 1980s and 1990s.

This strong commitment to mono-causalism in the case of AIDS meant that it was not permissible to draw on socio-economic explanations of disease (because the mono-causal account allows only for a single, typically microbial, source of disease to be included in the explanation). Without being able to draw on factors, such as poverty, in the explanation of disease, this rendered the disproportionate prevalence of AIDS in southern Africa somewhat mysterious.

## Was there really an explanatory gap in the case of AIDS?

This paper began with the concern that the transition from multiple theories of AIDS’s aetiology (some of which accommodated socio-economic factors) to the strictly viral account resulted in a loss of some explanatory power. In particular, some of the disproportionate prevalence of AIDS in southern Africa could not be accounted for in strictly viral terms but could be explained by appealing to socio-economic factors. If one is committed to strict mono-causalism about disease, which the AIDS research community in the 1990s was, then one cannot draw on non-viral factors in disease explanations. This section will look more closely at whether concerns about an explanatory gap created by too strict a commitment to mono-causalism are warranted in the case of AIDS.

At least three strictly viral accounts could be offered to explain the disproportionate prevalence of AIDS in southern Africa. First, it might be argued that there is a more virulent strain of HIV in southern Africa and that variation of prevalence tracks regional viral variation. Secondly, that southern Africans engage in riskier sexual practices (for instance, they might have greater numbers of partners), thus increasing the number of instances of possible exposure to disease.^4^ Third, that it is the practice of concurrent sexual partnerships, which is claimed to be more common in southern Africa than in other parts of the world, that explains the difference. The scientific evidence has resolutely rejected the first two of these possible explanations (UNAIDS 1999; Gray et al. 2001; Stillwaggon 2006). However, the third, the ‘concurrency theory’ has gained widespread acceptance and requires closer attention.

The central thesis of the concurrency theory is that there is a greater norm in southern Africa of individuals having a small number of long-lasting concurrent sexual relationships as opposed to the “serial monogamy that is more common in Western cultures” (Epstein 2007, 55) which explains the differences in prevalence. There is a plausible mechanism for this account, which is that individuals have increased viral loads shortly after infection and this, in turn, makes them more infectious during the “viremic window.” An individual who has concurrent sexual partnerships is more likely to pass on the virus to additional partners in the early stages infection than an individual engaged in serial monogamy who is less likely to have sexual contact with a new partner during the viremic window period (Epstein 2007, 61).

In addition to a plausible mechanism, the concurrency theory has gained support from the results of mathematical models, which show increased HIV prevalence in populations in which concurrency is modeled compared to those in which serial monogamy is modeled (Kretzschmar and Morris 1996; Morris and Kretzschmar 1997; Morris and Kretzschmar 2000). Indeed, a combination of the plausible mechanism and the mathematical modelling has made the concurrency theory the mainstream explanation for the disproportionate prevalence of HIV in southern Africa (Allais and Venter 2012; Sawers and Stillwaggon 2010).

However, on closer examination, the theory is less convincing than initially hoped. Much of the plausibility of the concurrency theory rests on the mathematical models, but the models themselves are based on implausible assumptions. Most implausibly, they assume that each individual involved in the concurrency network has sexual contact with all of his/her partners every day and that concurrency is gender symmetric (that is, that both men and women have multiple partners). The evidence supports neither of these assumptions, and once they have been adjusted to more closely align with data from the areas being analysed, the models fail to generate any difference in HIV prevalence between populations that practice concurrency and those that practice serial monogamy (Sawers and Stillwaggon 2010).

None of the strictly viral explanations are convincing, and the likelihood of contracting the virus in any particular instance of exposure remains important in the explanation of disproportionate prevalence.^5^ As noted before, the likelihood of contracting the virus in any particular sexual encounter is substantially higher in southern Africa than in the United States or Western Europe (Mosley 2004; Gray et al. 2001; Sanders and Sambo 1991). The mechanism for this is not mysterious—we are happy to accept that the poor are more susceptible to all sorts of other diseases because of poverty-induced diminished immune function (remember the example from Stillwagon about the introduction of the plague into Europe in the 1300s). In the case of AIDS, the mechanism from poverty to increased disease prevalence is that HIV positive individuals are more infectious when they have a high ‘viral load.’ Individuals who already have diminished immune function—due to malnutrition, concurrent exposure to other infections, etc. —spend more time at higher viral loads, thus making them more infectious for more of the time (Stillwaggon 2006).

A remaining question is why the mainstream AIDS research community in the 1990s was so committed to a mono-causal account of AIDS, given the compelling evidence for including socio-economic factors in the explanation of the disease. My own suspicion is that non-viral causal factors lost their explanatory credibility when HIV was accepted as the definitive cause of AIDS and had become too closely associated with the AIDS denialists— who continued to argue that AIDS is synonymous with diminished immune function resulting from malnutrition, STDs and drug use, to the exclusion of viral factors. To include too many socio-economic factors in one’s explanation was to risk aligning oneself too closely to the denialists.

Regardless of why the mono-causal account was so dominant in scientific AIDS research in the 1990s, this was the state of debate when Mbeki became President of South Africa in June 1999.

## South African AIDS denialism, and mono-causal and multi-causal theories

One plausible (but charitable) reading of the Mbeki case is that he was aware that there were aspects of the disease which the viral account did not explain. I will argue that this can be seen in his speeches on the subject in the late 1990s and early 2000s. In particular, that the viral account seemed incapable of explaining the disproportionate prevalence of AIDS in southern Africa, when compared to the United States or western Europe, leading Mbeki to consult scientists who strayed from the viral orthodoxy.

On this description of the Mbeki case, he began consulting non-mainstream AIDS scientists in order to find answers to the unresolved questions that he had identified, especially related to the regional variation of the disease, and the apparent connection between poverty and AIDS. Phrased slightly differently, the strictly mono-causal account of AIDS did not resolve issues that a more multi-causal account might have been able to, because a more multi-causal approach would have been able to draw on socio-economic factors in its explanation.

Mbeki’s speeches, from this period, add plausibility to the idea that this was his motivation. His opening speech at the 13th International AIDS Conference in Durban, on 9 July 2000, is particularly instructive in this regard. Much of this speech focuses on the impact of poverty on health in Africa. In conclusion to the discussion on poverty, he states that:

One of the consequences of this crisis [poverty] is the deeply disturbing phenomenon of the collapse of the immune system among millions of our people, such that their bodies have no natural defence against attack by many viruses and bacteria. (2000a)

This seems like an obvious reference to the immune overload theory of AIDS; poverty causes the immune system to collapse, which makes individuals vulnerable to opportunistic infections. Further, the idea that Mbeki turned to non-mainstream AIDS scientists because there were gaps in the mainstream viral research programme is alluded to again in the same speech when he states:

Some in our common world consider the questions I and the rest of our government have raised around the HIV-AIDS issue, the subject of the conference that you are attending, as akin to grave criminal and genocidal misconduct. What I hear being said repeatedly, stridently, angrily, is—do not ask questions! ... As I listened to the whole story being told about our country, it seemed to me that *we could not blame everything on a single virus.* (2000a, italics added)

The above quote, particularly the portion in italics, suggests that Mbeki was consulting non-mainstream scientists because he believed that there were aspects of the disease that required reference to concepts beyond the virus. Further support is added to this explanation of Mbeki’s action by comments in his welcome address to the members of the Presidential Panel (the advisory panel that he established which included both mainstream and non-mainstream AIDS scientists). This speech begins with Mbeki quoting AIDS prevalence statistics from a then recent World Health Organisation (WHO) report, particularly that sub-Saharan Africans make up “85% of the global total [of people diagnosed with AIDS], even though only one-tenth of the world population lives in sub-Saharan Africa” (Mbeki 2000b). This is the first suggestion that Mbeki was suspicious about the distribution of the disease. He then goes on to note that both the prevalence and the distribution of AIDS have changed in Sub-Saharan Africa but not in the US or Europe. He takes this to be a strange outcome, and one that requires further investigation, which had not at that point been undertaken within the viral research programme. In Mbeki’s words:

The situation has not changed in the United States up to today, nor in Western Europe with regard to homosexual transmission. But here [in southern Africa] it changed radically in a short period of time and increased radically in a short period of time. Why? This is obviously not an idle question for us because it bears very directly on this question: How should we respond? (2000b)

In this speech he also explicitly states that the reason the Presidential Panel had been assembled (and presumably the reason why non-mainstream AIDS scientists had been included on the panel) was to resolve these concerns. In this regard, Mbeki states that:

It is truly our hope that this process will help us to get to some of the answers, so that as public representatives we are able to elaborate and help implement policies that are properly focused, and that actually have an effect. I'm quite certain that *given the people who are participating in this panel*, we will get to these answers. (2000b, italics added)

These excerpts from Mbeki’s speeches indicate that it is plausible that his position might be at least partially explained by reference to the divide between mono-causal and multi-causal accounts of disease, and the resultant emphasis that this placed on viral aspects of AIDS research to the exclusion of socio-economic factors.

Other writers on Mbeki’s denialism have made similar suggestions. Butler and Mosley both argue that the mono-causal/multi-causal divide might have underpinned Mbeki’s denialism (although they each use slightly different language to make this point). Butler (2005) remains neutral about the relative merits of these perspectives and focuses instead on describing the context that allowed for the ascendancy of the multi-causal approach in South Africa. Mosley (2004) argues that the mono-causal and multi-causal approaches are both legitimate perspectives to adopt toward disease. As such, he concludes that Mbeki’s decision to go with a more multi-causal approach might have been justified because he made use of a legitimate alternative approach to thinking about disease.

Van Rijn (2006) makes a similar point to Mosley, suggesting that ‘virological’ (mono-causal) and ‘epidemiological’ (multi-causal) perspectives both provide legitimate ways of viewing disease and that Mbeki just happened to favour a more epidemiological approach. Further, Van Rijn seems to approve of Mbeki’s decision because he argues that Mbeki moved the discussion about AIDS beyond purely viral concerns, which is taken to be a good thing. Van Rijn is unclear about what aspect of Mbeki’s position he supports—presumably he means the inclusion of non-mainstream scientists on his advisory panel (given that this would have shifted the debate) and not the policy decision of making ARVs unavailable (given that this was due to a rejection of the causal role the virus played and did not just make the debate more inclusive of non-viral accounts).

Fassin (2007) similarly suggests that Mbeki’s position might be explained by reference to the division between ‘viral’ (mono-causal) and ‘sociological’ (multi-causal)^6^ accounts of the disease (15). However, he does not adopt Bulter’s value-neutral perspective on the division nor does he agree with Mosley that either approach is legitimate. He also does not follow Van Rijn’s suggestion that Mbeki productively advanced the discussion about AIDS beyond its purely viral elements. Instead, Fassin argues that the Mbeki case should put pressure on these underlying accounts of disease, such that an attempt should be made to unify the mono- and multi-causal approaches, stating that “one seeks a kind of third way, a means of making biological and social theories compatible” (15). I am sympathetic to Fassin’s position, and much of the rest of this paper will focus on trying to figure out what unifying these accounts would amount to.

## An attempt to resolve the mono-causal/multi-causal divide

At this point we have seen that there are two prominent accounts of disease on offer: the mono-causal account, which emphasises infectious microbially-based diseases, and the multi-causal account, as typified by the ‘web of causation.’ We have also seen that it is unclear what the relationship between the mono-causal and multi-causal accounts of disease is or should be, and that this is a source of theoretical as well as practical concern, as illustrated by the Mbeki case. Although some cursory points have already been made about this issue, in this section I will look more closely at this relationship.

There are at least three plausible descriptions of this relationship: 1) the mono-causal and multi-causal accounts are just different approaches to explain different sorts of diseases; 2) the multi-causal account subsumed the mono-causal account, and the microbial sources of disease were placed on the causal web as one type of cause amongst many; and 3) the multi-causal account of disease subsumed the mono-causal account, but the causal salience of microbial causes was preserved. Each of these will be discussed in turn.

### 1) Different accounts for different diseases

One way of dealing with the fact that there are two theories of disease is to argue that they are just different approaches for explaining different sorts of diseases: the mono-causal account is used to explain infectious, typically microbial diseases and is restricted to the realm of the biomedical bench sciences (virology, immunology, microbiology, etc.), and the multi-causal account, which explains CNCDs and is most closely linked to epidemiology. Russo suggests this as a possible interpretation when she states that: “[D]isease causation may be properly described by the mono-causal or the multi-causal model, but that depends on the disease at hand…” (2011, 75–76). This position is also implied by Mosley (2004) and Van Rijn (2006) in the literature on Mbeki’s denialism.^7^ I suspect that this way of thinking about the relationship between accounts of disease is the most descriptively accurate, or at least the one that best captures the way scientists were thinking about AIDS in the late 1980s and the 1990s. There are, however, a number of problems that result from adopting this approach.

The most obvious problem is that it is unclear how one should deal with diseases that are clearly infectious but for which socio-economic factors are causally salient. This is an issue not only for explaining the aetiology of AIDS but is also a concern for dealing with any disease that involves both microbial and social components, such as cholera or TB. Given the historical dominance of the mono-causal approach (Krieger 1994), the microbial aspects of disease are likely to take priority and maybe rightly so. However, if one is committed to the mono-causal and multi-causal accounts of disease as strictly separate, then this results in the exclusion of potentially relevant socio-economic factors.

Russo (2011) alludes to a related issue. A large part of the problem of ignoring socio-economic factors in order to be fully mono-causal about disease is that non-microbial causes are ignored for the purposes of health policy development (90–91). It is, however, obviously not the case that being mono-causal about disease will result in socio-economic factors being ignored completely in policy, but such concerns would be pursued for reasons external to the disease in question. If one is seriously committed to mono-causalism, then one cannot appeal to the role that socio-economic factors play in any particular disease in order to pursue policies to improve socio-economic conditions.

One might also be concerned that adopting a strictly mono-causal approach toward infectious diseases, especially in cases where it seems that socio-economic factors are actually at play, would leave one with an incomplete picture of the disease at hand. This is conceptually unsatisfying.

### 2) The multi-causal account of disease subsumes the mono-causal account

Broadbent (2009, 2013) suggests that the multi-causal account of disease subsumed the mono-causal account, providing a second way of thinking about the relationship between mono-causal and multi-causal accounts of disease. That is, the multi-causal account replaced the mono-causal account, such that the previously mono-causal sources of disease were placed on the web of causation as one cause amongst many. The result is that the restrictions on the mono-causal account were merely removed, leaving us with only the multi-causal model. Broadbent describes this as follows:

A multifactorial approach could simply reject the strictures of the monocausal model, asserting that diseases have many causes... This is the *bare* multifactorial model: it consists of no positive assertions about disease causation, and places no restrictions on what causal structures are specific to disease. (2009, 306)

This resolves the concern that was present when the mono-causal and multi-causal accounts were strictly divided. Because (on that account) the mono-causal and multi-casual accounts were different descriptions of different types of disease, if one classed a disease as infectious and thus mono-causal, then one was unable to appeal to aspects of the multi-causal approach (such as socio-economic drivers of disease). By removing the ‘one cause’ restriction from the mono-causal account and placing infectious agents on the ‘web of causation,’ this problem no longer exists because one is able to appeal to microbial and socio-economic sources of disease within the same model.

However, this is an obviously unsatisfying view of disease because it fails to pick out the causal salience of microbial agents. Broadbent describes this problem: “There is no discrimination and no hierarchy among causes, no ‘primarily caused by’—just a ‘constellation’ of causes which may come together in one or more than one way to give rise to a case of a disease” (2013, 154–155). In the case of AIDS, this would mean that HIV is placed on the causal web along with all the other factors associated with the disease; such as poverty, malnutrition and migrant labour paths. However, this fails to pick out the fact that HIV is more causally important to AIDS than the socio-economic drivers of the disease. We do not think that poverty is connected to AIDS in the same way as HIV (eliminating HIV would end AIDS but eliminating poverty would not). Our model of disease should make this distinction clear.

### 3) A layered multi-causal account of disease

Given the preceding discussion, what we want is an account of disease that unifies the mono-causal and multi-causal accounts, so that we can draw on explanatory tools from both but which preserves the causal salience of microbial agents. Susser’s (1973) multi-level, and multi-causal, model is successful on both counts. Susser maintains that our account of disease should be multi-causal but suggests that the central metaphor of the ‘web of causation’ be replaced with one of nested levels. The levels are determined by “systems,” where a system is:

… [A] set or assembly of factors connected with each other in some form of coherent relationship. A system is an abstraction. It allows a set of related factors to be described in terms of coherent structure or coherent function. (48)

Examples of systems include the cardiovascular system, made up of the heart and blood vessels; the body made up of many subsidiary biological systems; or a society made of many bodies. Part of what defines a system, on this account, is that coherent analysis can occur within its structural limits. For instance, a cardiologist might (hypothetically) be able to study the cardiovascular system by restricting her analysis to just its component parts without recourse to other systems (such as the external political system) (Susser 1973, 48).

Despite the fact that each system is a conceptually self-contained unit, they are layered in a nested fashion and different systems interact. For example, smoke in the environmental system might damage the blood vessels in the cardiovascular system. Studies can therefore be conducted on either one of two axes—they can be horizontal, when analysis occurs entirely within a single system (the cardiologist conducts a horizontal study when she studies just the components of the cardiovascular system), or they can focus on the vertical axis, in which case the analysis cuts across multiple systems (an epidemiologist conducting a study on AIDS might pursue a vertical study looking at multiple systems at once—the immune system, the social system, etc.). Importantly, whether a study is horizontal or vertical, and which systems are assessed, depends on the subject of study (Susser 1973, 49–50).

The distinction between horizontal and vertical studies becomes relevant for Susser’s view of causation. On his account, causation can either be ‘direct’ or ‘indirect.’ Direct causation occurs when cause and effect operate within the same system level (1973, 51). For instance, the calcified artery caused the heart to fail—the artery and the heart failure both occur within the cardiovascular system. Indirect causation occurs when cause and effect operate at different system levels. For instance, prolonged smoking causes the artery to calcify, which in turn causes the heart to fail. Smoking is an indirect cause of the heart failure because the smoking and the heart failure occur within different system levels (smoking might occur at the behavioural level, while heart failure occurs at the level of the cardiovascular system).^8^

Susser’s account therefore allows one to appeal to both microbial sources of disease and socio-economic factors within a single account because both are included in the same model. Further, in the case of HIV, it picks out the causal salience of the virus because both the virus and the T-Cells that are targeted by the virus exist at the same system level, making the virus the direct cause of the disease. Thus, Susser’s account resolves both problems previously identified: it unifies the mono-causal and multi-causal accounts of disease, and it allows for certain causes to be picked out as salient. Russo (2011) maintains that Susser’s account is a clear improvement on the ‘bare-multifactoralism’ of the web of causation but also creates some philosophical concerns through its introduction of the notion of levels (68).

## Some philosophical problems with levels

Drawing on “levels” to resolve the divide between mono-causal and multi-causal accounts of disease introduces problems because, as Craver (2007) points out, “the term ‘level’ is multiply ambiguous” (163). In the special sciences, one can mean a number of different things when talking about levels:

[T]here are levels of abstraction, analysis, behavior, complexity, description, explanation, function, generality, organization, science, and theory. Consequently, scientific and philosophical disputes about levels cannot be addressed, let alone resolved, without first sorting out which of the various senses of “level” is under discussion. (164)

Given that Susser specifies that the content of his levels are “systems,” where a system is defined by a number of component parts operating together to fulfil a specific function (1973, 48), it might seem clear that he takes levels to be functional. However, he goes on to state that “a system is an abstraction” (48), which makes it seem as though he considers the levels in his account to be levels of abstraction. He later argues that different systems/levels are the subject of different academic disciplines, which makes it seem as though his levels are organised around what Craver terms the ‘products of science’ (2007, 171). There is, therefore, ambiguity surrounding the ontological status of Susser’s levels. Also unclear is the nature of the relationship between the levels. In other words, Susser tells us that different levels can interact with each other but not much else.

Should we worry that Susser does not provide us with an ontologically satisfying account of the layers that underpin his account? I do not think so. As Broadbent points out (in a completely unrelated debate), we can distinguish between good metaphysics/ontology and good methodology, and when the stakes are high (as they obviously are in the case of AIDS), we might favour good methodology. In fact, sometimes it would be irresponsible to go with a good metaphysical/ontological account to the detriment of methodology (Broadbent 2011, 65). Accepting Broadbent’s point that good methodology can sometimes trump good metaphysics, we should favour Susser’s account because it effectively unifies the mono-causal and multi-causal theories of disease, while allowing us to pick out certain factors as causally salient.

## Conclusion

In this paper, I have argued that there is some ambiguity surrounding the relationship between mono-causal and multi-causal accounts of disease (in both the descriptive and the normative sense). Furthermore, this confusion might help us better understand Thabo Mbeki’s AIDS denialism in South Africa in the early 2000s. Given the scale of the tragedy in the South African case, this should put pressure on us to find a unified account of the mono-causal and multi-causal accounts of disease, one which allows us to pick out the causal salience of particular factors. I conclude that Susser’s layered and nested account allows us to do both and that it should be accepted as methodologically sound, even if metaphysically troubling. The main benefit to emerge from the more unified causal understanding, particularly as we enter the post-AIDS era, is that it provides us with the conceptual tools to underpin AIDS interventions that deal with socio-economic and viral aspects of the disease simultaneously. This paper began with Whiteside’s observation that the diminishing importance of AIDS on the international agenda is at least partially driven by the misconception that excellent technical knowledge of the viral aspects of the disease will end the epidemic. Having a better understanding of the relationship between the mono-causal and multi-causal aspects of the disease should put an end to this misconception.

### Endnotes

^1^ Gray et al. (2001), Sanders and Sambo (1991) and UNAIDS (1999) make similar points.

^2^ Mbeki’s “AIDS denialism” refers to his support of non-mainstream AIDS scientists, and his policy of refusing to provide antiretroviral therapy (the drugs needed to treat AIDS) via the South African public health system. The non-mainstream scientists that Mbeki supported denied the causal connection between HIV and AIDS.

^3^ It should be noted that Lee (2012) and Broadbent (2013) both point out that the germ theory of disease and the mono-causal account are conceptually distinct. One can be committed to the mono-causal account without being committed to the single cause of any particular disease being microbial. Diseases of deficiency provide good examples of this. For instance, scurvy can be explained mono-causally by reference to a Vitamin C deficiency. However, historically, those endorsing the mono-causal account of disease have largely focussed on microbial agents.

^4^ Significantly, any socio-economic account that amounts to an explanation of why individuals or groups have increased instances of exposure to possible infection will be subject to explanation via the strictly viral account of disease. For instance, descriptions that draw on elements of the South African mining sector to explain why individuals might engage in riskier sexual practices are easily accommodated within the mono-causal model, because (biologically speaking) it just amounts to an increased number of possible instances of exposure.

^5^ This is not a claim about the poor having more or riskier sex. The issue here is not how many instances of contact there are, but how likely one is to contract the virus in any particular instance of sexual contact.

^6^ I am aware that “mutli-causal” and “sociological” are not synonymous. However, given the emphasis on microbial sources of disease in the mono-causal account, and the idea that any account of disease that is not mono-causal (in the standard sense of being concerned with microbes) is multi-causal, it seems that Fassin is pointing to a multi-causal approach to thinking about AIDS when he uses the term “sociological.” His overall point here is that we need to think about AIDS in a way that takes account of viral and non-viral drivers of disease at the same time.

^7^ However, the relationship between mono-causal and multi-causal theories of disease is not the primary concern of these authors, and so this is at least partly read into their accounts. If asked directly, they might not explicitly endorse this view of the relationship between the mono-causal and multi-causal accounts.

^8^ This is just an example to illustrate the point. It is not meant to be an accurate description of cardiology or the causes of heart attacks.
